# T cells isolated from G-CSF-treated multiple myeloma patients are suitable for the generation of BCMA-directed CAR-T cells

**DOI:** 10.1016/j.omtm.2022.06.010

**Published:** 2022-06-22

**Authors:** Anthony M. Battram, Aina Oliver-Caldés, Maria Suárez-Lledó, Miquel Lozano, Miquel Bosch i Crespo, Núria Martínez-Cibrián, Joan Cid, David F. Moreno, Luis Gerardo Rodríguez-Lobato, Alvaro Urbano-Ispizua, Carlos Fernández de Larrea

**Affiliations:** 1 Institut d'Investigacions Biomèdiques August Pi i Sunyer (IDIBAPS), 08036 Barcelona, Spain; 2 Department of Hematology, Amyloidosis and Myeloma Unit, Hospital Clínic of Barcelona, 08036 Barcelona, Spain; 3 Apheresis & Cellular Therapy Unit, Department of Hemotherapy & Hemostasis, ICMHO (Institut Clínic de Malalties Hematològiques i Oncològiques), Hospital Clínic of Barcelona, University of Barcelona, 08036 Barcelona, Spain; 4 Josep Carreras Leukaemia Research Institute, 08036 Barcelona, Spain; 5 Department of Haematology, University of Barcelona, 08036 Barcelona, Spain

**Keywords:** CAR-T cells, G-CSF, multiple myeloma, BCMA, ASCT, ARI0002

## Abstract

Autologous cell immunotherapy using B cell maturation antigen (BCMA)-targeted chimeric antigen receptor (CAR)-T cells is an effective novel treatment for multiple myeloma (MM). This therapy has only been used for relapsed and refractory patients, at which stage the endogenous T cells used to produce the CAR-T cells are affected by the immunosuppressive nature of advanced MM and/or side effects of previous therapies. An alternative pool of “fitter” T cells is found in leukocytoapheresis products that are routinely collected to obtain hematopoietic progenitor cells for autologous stem cell transplantation (ASCT) early in the treatment of MM. However, to mobilize the progenitor cells, patients are dosed with granulocyte colony-stimulating factor (G-CSF), which is reported to adversely affect T cell proliferation, function, and differentiation. Here, we aimed to first establish whether G-CSF treatment negatively influences T cell phenotype and to ascertain whether previous exposure of T cells to G-CSF is deleterious for anti-BCMA CAR-T cells. We observed that G-CSF had a minimal impact on T cell phenotype when added *in vitro* or administered to patients. Moreover, we found that CAR-T cell fitness and anti-tumor activity were unaffected when generated from G-CSF-exposed T cells. Overall, we showed that ASCT apheresis products are a suitable source of T cells for anti-BCMA CAR-T cell manufacture.

## Graphical abstract

**Figure:**
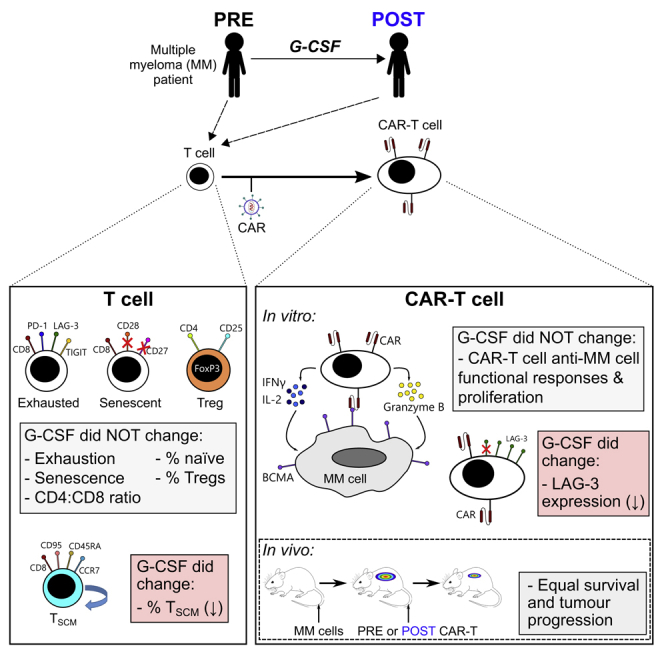

## Teaser

Battram et al. show that BCMA-targeting chimeric antigen receptor (CAR)-T cells generated from T cells exposed to G-CSF *in vivo* have a favorable phenotype and effective anti-tumor activity, thus demonstrating that apheresis products collected for autologous stem cell transplantation are a suitable source of T cells for CAR-T cell production.

## Introduction

Multiple myeloma (MM) is a B cell malignancy that accounts for almost 1% of all newly diagnosed cancers.^1^ Although treatment options for MM have undergone radical improvements over recent decades, the disease remains incurable, and relapse is inevitable in almost all cases.^2^ Chimeric antigen receptor (CAR)-modified T cells that target B cell maturation antigen (BCMA) have revolutionized MM therapy and are likely to become a standard part of the treatment regimen for this hematological cancer.^3^^,^^4^ Indeed, the anti-BCMA CAR-T cell product idecabtagene vicleucel became the first cell-based immunotherapy indicated for the treatment of relapsed and refractory (R/R) MM that was approved by the US Food and Drug Administration (FDA) and European Medicines Agency (EMA),^5^ and many others are currently being investigated in clinical trials.^6^^,^^7^

Presently, most developed CAR-T cells are autologous therapies that are made by transducing a CAR molecule into endogenous T cells derived from the patient that is destined to receive the treatment.^8^ For MM, current clinical trials for anti-BCMA CAR-T cells have recruited patients who have relapsed multiple times in response to several therapeutic agents, including the three main families of drugs for this disease; proteasome inhibitors (bortezomib and carfilzomib), immunomodulatory drugs (lenalidomide and pomalidomide), and anti-CD38 monoclonal antibodies.^9^ However, at this stage in the disease, T cell abnormalities are promoted by cytokines secreted by malignant plasma cells and suppressive immune cells as well as other thus far uncharacterized mechanisms.^10^, ^11^, ^12^ Furthermore, previous lines of therapy can have adverse consequences for T cell immunity, such as dexamethasone-mediated abrogation of T cell proliferation and depletion of naive CD8^+^ T cells by the anti-CD38 antibody daratumumab.^13^, ^14^, ^15^ This undesirable combination of disease- and drug-induced effects results in an immunosuppressive microenvironment and T cell deficiency, which manifests itself as exhaustion, senescence, loss of early-differentiated cells, an alteration in the CD4:CD8 ratio, and metabolic dysfunction.^11^^,^^14^^,^^16^, ^17^, ^18^ Analysis of the parameters that influence the efficacy of one anti-BCMA CAR-T cell product revealed that some of these factors, particularly fewer CD8^+^ early differentiated (CD45RO^−^CD27^+^) cells and a lower CD4:CD8 ratio in the starting T cell product, were associated with a poorer clinical response.^19^ Taken together, these studies clearly indicate that, for CAR-T cell generation, it would be beneficial to use T cells isolated earlier in the treatment program, before a refractory disease develops.

An alternative and readily available source of “fitter” T cells are the apheresis products that are routinely collected and cryopreserved when harvesting progenitor cells for autologous stem cell transplantation (ASCT), which is normally conducted early in the treatment plan of MM patients.^20^ To mobilize progenitor cells from the bone marrow into the peripheral blood ready for collection, patients are administered with granulocyte colony-stimulating factor (G-CSF).^21^ Either directly or indirectly, G-CSF is proposed to have negative effects on conventional T cells, such as reducing proliferation,^22^ impairing CD8^+^ T cell effector function,^23^^,^^24^ causing an imbalance in the ratio of Th1/Th2 cells,^25^, ^26^, ^27^ and inducing regulatory T cell (Treg) expansion.^28^ Such impairments could reduce the effectiveness of CAR-T cells that are generated from G-CSF-exposed T cells, but this is currently unknown. Moreover, apheresis products are replete with myeloid cells, including CD14^+^ monocytes, which are known to limit CAR-T cell expansion.^29^^,^^30^

In this report, we studied whether G-CSF adversely affected freshly isolated T cells, with particular attention paid to characteristics that had previously been associated with anti-BCMA CAR-T cell clinical responses. Next, anti-BCMA CAR-T cells were generated from T cells previously exposed to G-CSF *in vivo* and the expanded CAR-T cells were assessed for phenotype and functionality. The BCMA-targeting CAR molecule used in this study is a fully academically developed CAR called ARI0002h (henceforth referred to as ARI2h),^31^^,^^32^ which is currently being explored in a clinical trial for patients with R/R MM (CARTBCMA-HCP-01 trial, NCT04309981). Overall, we found that the impact of G-CSF was minimal and, most importantly, did not diminish CAR-T cell activity.

## Results

### Recombinant G-CSF had no direct effect on ARI2 h expansion or activity

**Figure 1:**
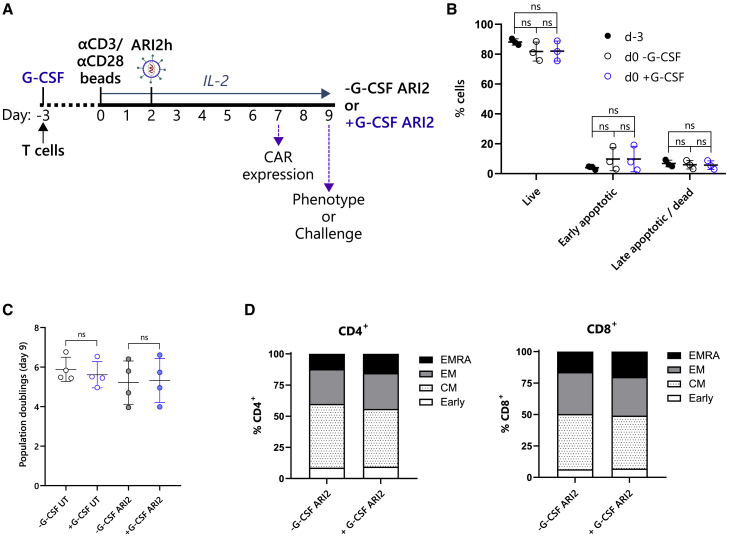
ARI2 h expansion and phenotype are unaffected by recombinant G-CSF (A) Schematic of cell culture protocol for production of ARI2h cells with (+G-CSF ARI2) or without (−G-CSF ARI2) exposure to 10 ng/mL G-CSF. (B) Cells were stained with annexin V (AnV) and 7-AAD on day −3 (d−3) before exposure to G-CSF and afterward on day 0 (d0) to determine the frequency of live cells (AnV^−^7-AAD^−^), early apoptotic cells (AnV^+^7-AAD^−^), and late apoptotic or dead cells (AnV^+^7-AAD^+^). See also Figure S2C. (C) Growth of untransduced (UT) and ARI2h cells was measured as number of population doublings on day 9 compared with day 0. (D) Summary of memory and effector phenotypes of expanded CAR^+^ ARI2h cells is shown, categorized as early differentiated (early, CCR7^+^CD45RA^+^), central memory (CM) (CCR7^+^CD45RA^−^), effector memory (EM) (CCR7^−^CD45RA^−^), and effector memory CD45RA^+^ (CCR7^−^CD45RA^+^) cells. Data represent the mean percentage of each population. n = 3. All error bars show mean ± SD. ns, not significant.

Although many of the inhibitory actions of G-CSF on T cells are secondary effects caused by the regulation of other cell types, it has been reported that G-CSF directly controls T cell function.^25^^,^^33^ To examine whether these direct effects impact anti-BCMA CAR-T cell activity, T cells were cultured for 3 days in the presence of recombinant G-CSF prior to activation, ARI2h CAR transduction, and cell expansion (Figure 1A). The efficacy of the recombinant G-CSF was tested by analyzing G-CSF-induced STAT3 phosphorylation in monocytes (Figure S1).^34^ There was no loss in T cell viability following the initial pre-stimulation incubation stage, and importantly, the addition of G-CSF did not impair T cell survival or induce apoptosis (Figures 1B and S2). At the end of the ARI2h production, it was found that pre-incubation with G-CSF had no effect on cell growth in terms of either population doublings or differentiation (Figures 1C and 1D).

**Figure 2:**
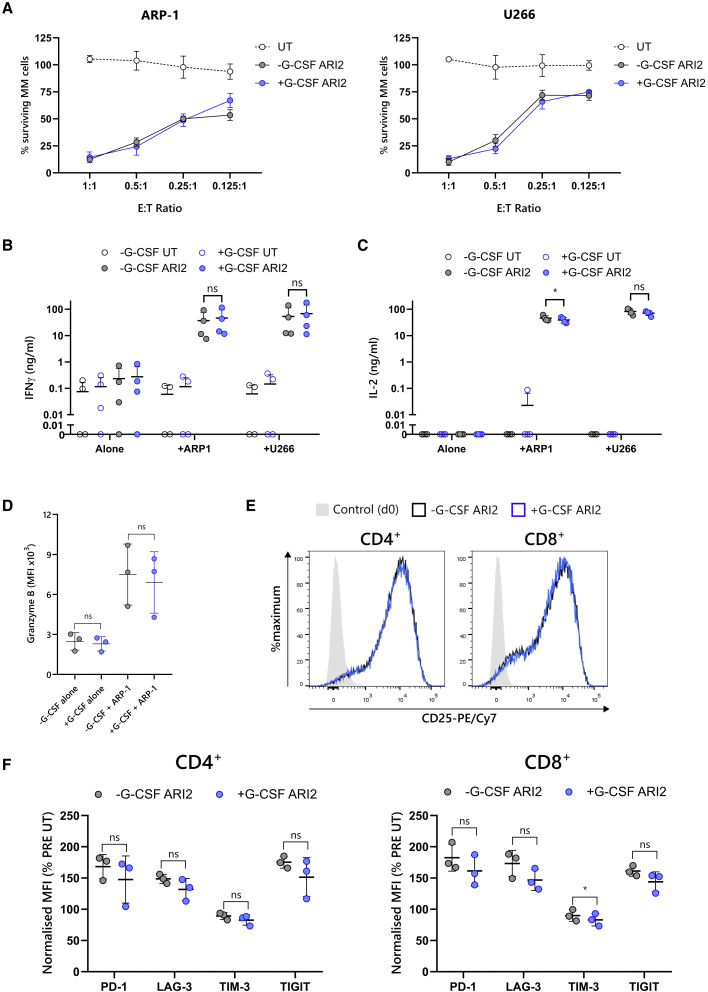
Recombinant G-CSF does not diminish ARI2 h activity or promote ARI2 h exhaustion (A) Survival of GFP-ffLuc-expressing ARP-1 (left) or U266 (right) myeloma cell lines following a 24-h co-culture with UT or ARI2h cells at the indicated T cell:tumor cell line (effector:target [E:T]) ratios, determined by measuring target cell bioluminescence (n = 4). Data are represented as mean ± SEM. (B–D) UT and ARI2h cells were co-incubated with ARP-1 cells, U266 cells, or media alone (alone) for 6 h. Levels of IFNγ (B) and IL-2 (C) released into the supernatant were evaluated by ELISA, and the expression of granzyme B in CAR^+^ cells was assessed by flow cytometry, displayed as median fluorescence intensity (MFI) (D). Error bars show mean ± SD. (E) Histogram of CD25 expression on CAR^+^ CD4^+^ (left) and CD8^+^ (right) T cells and unstimulated day 0 T cells (control), representative of three experiments, is shown. (F) Summary of the surface expression of PD-1, LAG-3, TIM-3, and TIGIT on CAR^+^ CD4^+^ (left) and CD8^+^ (right) T cells is shown, represented as MFI normalized to that displayed on UT PRE G-CSF UT cells. Error bars show mean ± SD.∗p < 0.05; ns, not significant.

When ARI2h cells were challenged with two MM cell lines, ARP-1 and U266, it was observed that G-CSF had no impact on the anti-tumor killing ability of the CAR-T cells (Figure 2A). Correspondingly, G-CSF exposure had no bearing on tumor-cell-induced interferon γ (IFNγ) secretion (Figure 2B). Although there was a small difference in interleukin-2 (IL-2) release in response to ARP-1 (p = 0.047), the average reduction in IL-2 was small (6.6 ng/mL) in comparison to the total amount found in the supernatant, and no difference was observed with U266 cells (Figure 2C). Similarly, G-CSF did not alter ARI2h granzyme B levels, either basally or following stimulation with tumor cells (Figure 2D).

Another aspect of CAR-T cells that is thought to contribute to *in vivo* functionality is their expression of key activation and inhibitory molecules. After expansion, ARI2h CD4^+^ and CD8^+^ cells expressed high levels of CD25, the IL-2 receptor α chain (Figure 2E), which is used to measure CAR-T cell activation through IL-2 signaling but can also demonstrate tonic signaling when above normal levels.^35^ G-CSF did not alter ARI2h CD25 expression, suggesting that G-CSF neither decreased cell activation nor induced spontaneous tonic signaling. In addition, levels of checkpoint molecules PD-1, LAG-3, TIM-3, and TIGIT were not augmented by G-CSF; indeed, TIM-3 expression was reduced on CD8^+^ cells (Figure 2F).

Collectively, these data demonstrate that G-CSF did not exert any direct *in vitro* effects on T cells that subsequently impaired CAR-T cell proliferation, function, or phenotype.

### Patient G-CSF treatment did not alter the T cell CD4:CD8 ratio, increase the proportion of Tregs or induce T cell dysfunction

After establishing that G-CSF did not directly impact ARI2h cells *in vitro*, we explored the possibility of generating ARI2h cells from patients who received G-CSF to mobilize CD34^+^ progenitor cells prior to ASCT. All patients were diagnosed with MM, and ASCT was being used as part of a first-line therapy strategy following induction treatment (Table S1). The induction therapy used for all patients was similar both in terms of drugs administered and number of treatment cycles (Table S2).

**Figure 3:**
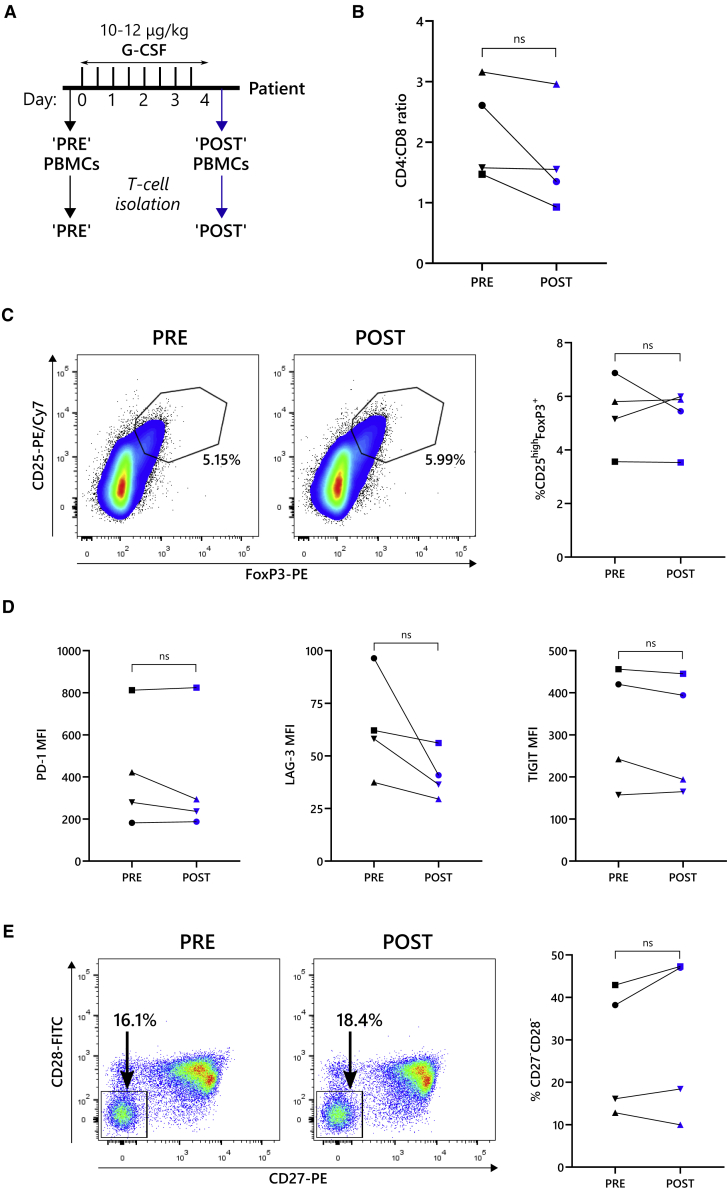
Patient populations of Tregs and dysfunctional T cells are not increased following G-CSF administration (A) Schematic of G-CSF dosing. Patients were administered with 10–12 μg/mL G-CSF every 12 h for a total of 4 days. Before (PRE) and after (POST) G-CSF treatment, PBMCs were harvested from the patients, from which T cells were isolated. (B) Ratio of CD4^+^ and CD8^+^ cells in freshly isolated T cells is shown. (C) Frequency of CD4^+^ CD25^high^FoxP3^+^ Tregs within the PRE G-CSF and POST G-CSF samples is shown as FACS plots (left) and summarized as a percentage of total CD4^+^ T cells (right). Numbers on FACS plots indicate the percentage of cells found within the Treg gate. (D) Expression of exhaustion markers PD-1 (left), LAG-3 (middle), and TIGIT (right) on PRE G-CSF and POST G-CSF CD8^+^ T cells is shown, displayed as MFI. (E) Senescent-like CD27^−^CD28^−^ cells were identified by flow cytometry and are presented as FACS plots (left) and summarized as a percentage of total CD8^+^ T cells (right). Numbers on FACS plots indicate the percentage of cells found within the CD27^−^CD28^−^ gate. Data points in (B)–(E) are coded according to patient donor and are consistent with other figures. ns, not significant.

To investigate the impact of G-CSF on CAR-T cells generated from MM patient T cells, first it was necessary to study the potential effects of G-CSF on T cells before CAR-T cell generation. As such, blood was collected from patients before (“PRE”) and after (“POST”) 4 days of G-CSF treatment (10–12 μg/kg every 12 h) and subject to analysis of T cell populations and phenotype (Figure 3A). During the T cell isolation step, monocytes were carefully removed to avoid potential issues with CAR-T cell expansion (Figure S3).^29^ From the isolated T cells, the relative proportion of CD4^+^ and CD8^+^ T cells varied dramatically (0.93–3.16), as previously described for MM patients,^18^ but there was no significant difference in the CD4:CD8 ratio between PRE and POST samples (p = 0.1595) (Figure 3B). As G-CSF has been described to cause Treg expansion,^28^ the frequency of Tregs, characterized as CD25^high^FoxP3^+^ cells, was assessed. Tregs made up less than 7% of the total CD4^+^ T cells in all analyses, with no difference observed between PRE and POST samples (p = 0.794) (Figure 3C).

Another potential factor that could influence the quality of CAR-T cells is the dysfunctionality of the T cells in the starting material. To compare the exhaustion phenotype between PRE and POST CD8^+^ T cells, expression of the checkpoint molecules PD-1, LAG-3, and TIGIT were analyzed, but levels of these markers were similar (Figure 3D). TIM-3 expression was also evaluated but was too low to make accurate comparisons (data not shown). Another feature of T cell fitness is senescence, which can be characterized as the presence of CD27^−^CD28^−^ cells within the CD8^+^ pool.^36^ In some patients, senescent-like cells constituted more than 40% of CD8^+^ T cells, but this was consistent in both PRE and POST G-CSF (Figure 3E).

### *In vivo* G-CSF reduced frequency of T_SCM_ cells but had no effect on other effector and memory T cell populations

**Figure 4:**
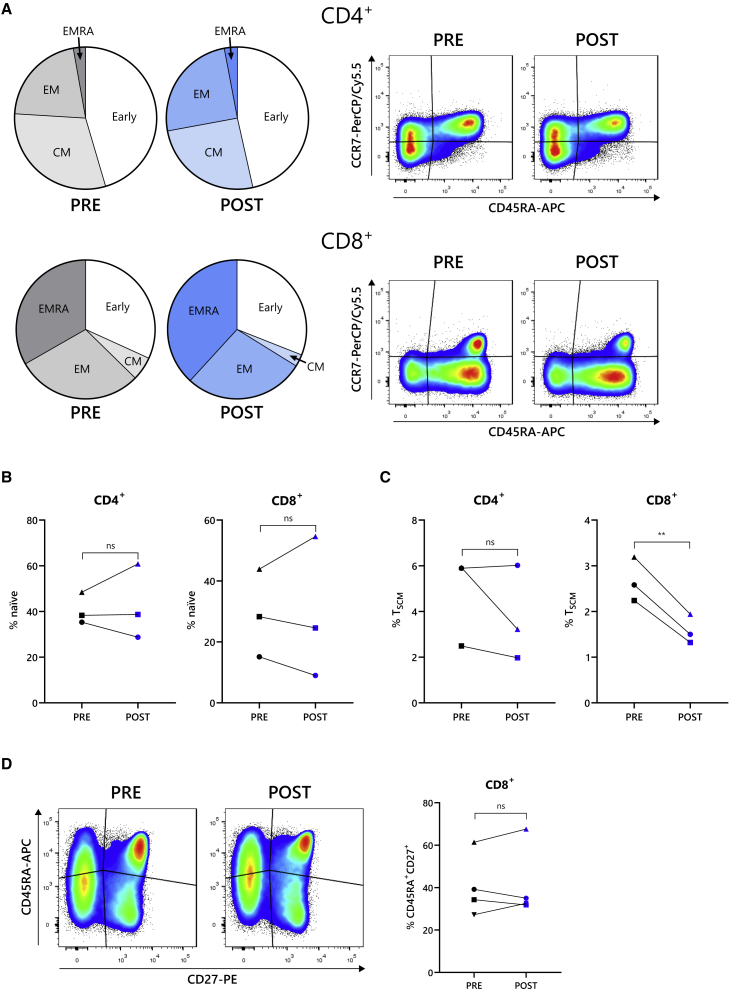
Presence of stem cell memory T cells diminished by G-CSF, but other memory and effector populations are unchanged (A) Memory and effector phenotypes of patient-derived CD4^+^ (upper row) and CD8^+^ (lower row) T cells before (PRE) and after (POST) G-CSF administration, categorized as early differentiated (early) (CCR7^+^CD45RA^+^), CM (CCR7^+^CD45RA^−^), EM (CCR7^−^CD45RA^−^), and effector memory CD45RA^+^ (CCR7^−^CD45RA^+^) cells. Summarized in pie charts (left) shows the mean percentage of each population and displayed as FACS plots. n = 4. (B and C) Measurement of CD95 expression on early differentiated CD4^+^ (left) and CD8^+^ (right) CCR7^+^CD45RA^+^ cells enabled the identification of CD95^−^ naive cells (B) and CD95^+^ stem cell memory T (T_SCM_) cells (C). Data are shown as a percentage of total CD4^+^ or CD8^+^ cells. (D) CCR7/CD45RA staining of patient T cells to identify the CCR7^+^CD45RA^+^ early differentiated population is shown. Data are displayed as example FACS plots (left) and summary of percentage of CCR7^+^CD45RA^+^ cells from all donors (right). Data points in (B)–(D) are coded according to patient donor and are consistent with other figures. ∗∗p < 0.01; ns, not significant.

The effector and memory phenotypes of T cells are another important determinant of their activity, and for anti-BCMA CAR-T cells, the presence of early differentiated memory cells within the starting T cell product correlates with patient outcomes.^19^ When comparing the frequency of effector and memory cell populations between PRE and POST CD4^+^ and CD8^+^ T cells, based on CD45RA and CCR7 staining, no difference was observed (Figure 4A). To further identify the presence of distinct memory subsets, the expression of CD95 was evaluated on “early” differentiated CD45RA^+^CCR7^+^ cells, which distinguishes between naive populations and those with sustained self-renewal potential, termed stem-cell-memory-like (T_SCM_) cells.^37^ Although the proportion of CD4^+^ and CD8^+^ naive (CD95^−^) cells was unchanged by G-CSF exposure (Figure 4B), the frequency of CD8^+^ T_SCM_ (CD95^+^) cells was 40% lower in POST G-CSF samples (p = 0.008) (Figure 4C).

An alternative approach to identify T cell memory phenotypes is to stain for CD45RA/CD45RO and CD27, which is particularly relevant to CAR-T cell production, as the presence of CD8^+^CD45RO^−^CD27^+^ cells in the starting material is a known predictor of patient outcomes.^19^^,^^38^ Analysis of CD8^+^CD45RA^+^CD27^+^ cells, which are equivalent to CD8^+^CD45RO^−^CD27^+^ cells,^39^ revealed that the proportion of this clinically important population was unaltered by G-CSF (Figure 4D).

Put together, the data from T cells before and after G-CSF administration demonstrate that G-CSF had only a minor impact on T cell phenotype and populations, with the only identified change being the proportion of the small pool of T_SCM_ cells.

### High expansion rates and increased T cell isolation after G-CSF treatment compensated for reduced CAR transduction

**Figure 5:**
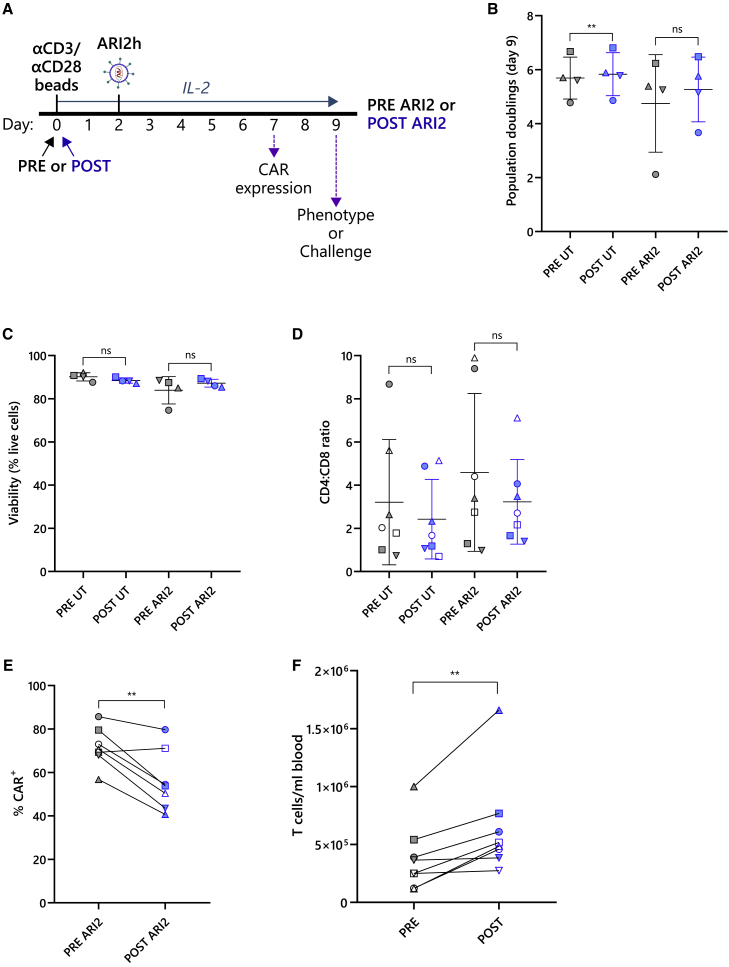
Decrease in CAR transduction of POST G-CSF T cells is compensated by an increase in the concentration of isolated T cells (A) Schematic of cell culture protocol for production of ARI2h cells from patient-derived T cells that were from before (PRE ARI2) or after (POST ARI2) G-CSF administration. (B) Growth of untransduced (UT) and ARI2 h cells measured as number of population doublings on day 9 compared with day 0 is shown. (C) UT and ARI2 h cells were stained with annexin V (AnV) and 7-AAD to assess viability, presented as percentage of live cells (AnV-7-AAD^−^). (D) Ratio of CD4^+^ and CD8^+^ cells in UT and CAR^+^ ARI2 h cells on day 9 is shown. (E) Percentage of PRE ARI2 and POST ARI2 cells that were positive for the ARI2 h CAR molecule on day 7 is shown. (F) Number of T cells isolated from the blood of patients before (PRE) and after (POST) G-CSF treatment is shown, normalized to the volume of blood drawn. All error bars show mean ± SD, and data points in (B)–(F) are coded according to patient donor and are consistent with other figures. ∗∗p < 0.01; ns, not significant.

After establishing that T cells from patients after G-CSF treatment were suitable for CAR-T cell production, ARI2h cells were expanded as shown in Figure 5A. Sufficient proliferation of ARI2h cells was observed regardless of whether the T cells had been exposed to G-CSF, with population doubling rates exceeding four for 75% of the PRE and POST cultures (Figure 5B). Interestingly, untransduced (UT) cells expanded from POST G-CSF T cells actually grew better than UT cells from PRE G-CSF T cells. The viability of UT or ARI2h cells was 85% or higher (Figure 5C), with the exception of one PRE ARI2h culture, which was still above the limit (70%) for satisfactory clinical production of ARI2h.^31^ Other parameters that are often important for CAR-T cell production are the CD4:CD8 ratio and the CAR transduction rate. In the majority of both the PRE and POST ARI2h cultures, CD4^+^ T cells outnumbered CD8^+^ T cells on day 7 of the expansion, but the CD4:CD8 ratio showed a high degree of variability between donors (0.7–9.9) (Figure 5D).

All CAR-T cell cultures achieved a CAR transduction efficiency that is suitable for clinical purposes,^31^ but PRE ARI2h cultures exhibited a higher percentage of CAR^+^ cells compared with the corresponding POST ARI2h cultures in six out of seven expansions (p = 0.006) (Figure 5E). However, it is worth noting that the number of T cells obtained from all patients after 4 days of G-CSF treatment was higher than before they had been given G-CSF (p = 0.008) (Figure 5F). This 2-fold increase in isolated T cells allows for the generation of more ARI2h cells from a given patient, despite the 22% reduction in transduction efficiency in POST ARI2h cultures.

In summary, POST ARI2h cells expanded as well as PRE ARI2h cells and, although there was a difference in transduction rates, this was offset by the fact that many more T cells are obtained from the same volume of blood following G-CSF administration.

### ARI2h cells generated from PRE and POST T cells demonstrate equal anti-tumor activity *in vitro* and *in vivo*

**Figure 6:**
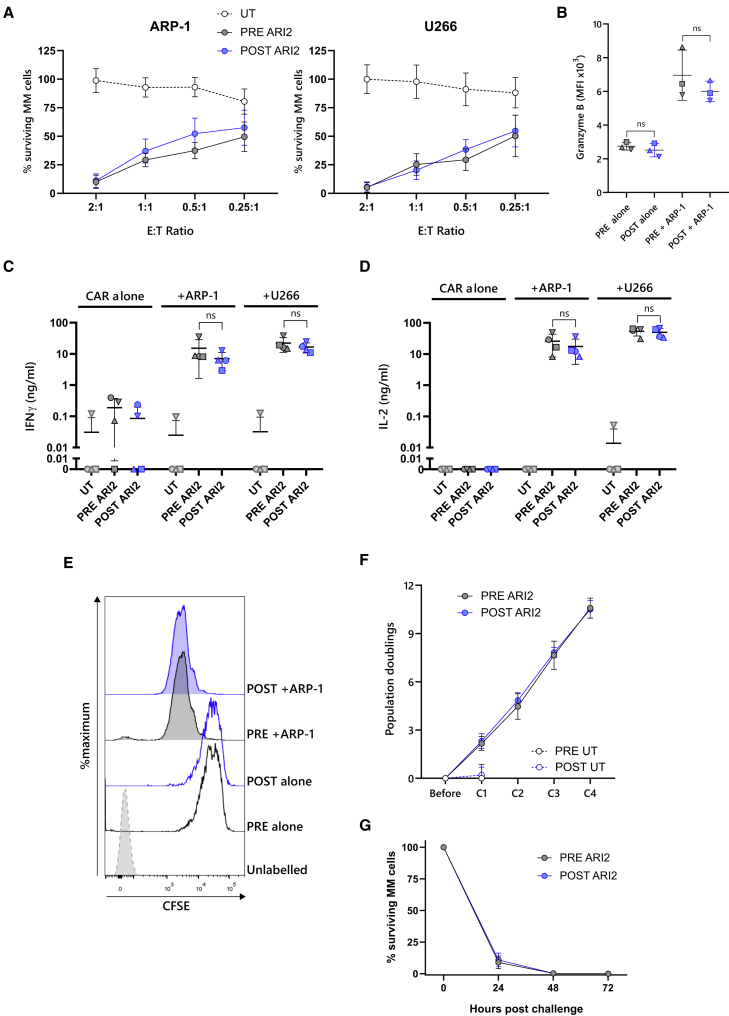
POST G-CSF ARI2h anti-myeloma cell responses are effective (A) Survival of GFP-ffLuc-expressing ARP-1 (ARP-1-GFP-ffLuc) (left) or U266 (right) myeloma cell lines following a 24-h co-culture with UT or ARI2h cells at the indicated T cell:tumor cell line (effector:target [E:T]) ratios, determined by measuring target cell bioluminescence (n = 4 to 5). Data are represented as mean ± SEM. (B–D) UT and ARI2 h cells were co-incubated with ARP-1 cells, U266 cells, or media alone (CAR alone) for 6 h. The expression of granzyme B in CD8^+^ CAR^+^ cells was assessed by flow cytometry (B), and levels of IFNγ (C) and IL-2 (D) released into the supernatant were evaluated by ELISA. Error bars show mean ± SD. Data points are coded according to patient donor and are consistent with other figures. See also Figure S4A. (E) ARI2h cells were labeled with CFSE and cultured with (+ARP-1) or without (alone) ARP-1 cells. After 72 h, ARI2 h cell proliferation was analyzed by measuring CFSE dilution. ARI2h cells that were not treated with CFSE (unlabeled) acted as a control. The displayed histograms are CD8^+^ CAR^+^ cells and are representative of three experiments. See also Figure S4B. (F and G) Fold expansion of ARI2h cells following four challenges (C1, C2, C3, and C4) with ARP-1-GFP-ffLuc cells at a 0.125:1 E:T ratio (n = 3; F) is shown. Survival of ARP-1-GFP-ffLuc cells during the fourth stimulation is shown, determined by measuring target cell bioluminescence (n = 3) (G). Data are represented as mean ± SEM. ns, not significant. See also Figure S5.

To determine whether G-CSF affected POST ARI2h cell functional ability, PRE and POST ARI2h cells were challenged with MM tumor cell lines and their responses were analyzed. Both PRE and POST ARI2h cells exerted a similar cytotoxicity against MM cells and secreted equal levels of effector molecules, including granzyme B, IFNγ, and IL-2 (Figures 6A–6D and S4A). Furthermore, the proliferative responses of PRE and POST ARI2h cells to MM cell line ARP-1 were equivalent (Figures 6E and S4B).

To test the persistence of PRE and POST ARI2h cell responses, they were repeatedly stimulated with ARP-1 cells *in vitro* and subsequently assessed for their ability to expand and kill the tumor cells (Figure S5A). For a period of four consecutive challenges, PRE and POST ARI2h cells exhibited similar large-scale expansion (average doubling rates of 10.58 and 10.51 for PRE ARI2h and POST ARI2h, respectively) and rapid tumor cell killing that was maintained with each stimulation (Figures 6F, 6G, and S5B). After the repeated stimulations, almost all T cells in the PRE and POST ARI2h cell cultures were CAR^+^ (Figure S5C), with a similar differentiation status and expression of exhaustion-associated inhibitory receptors (Figures S5D–S5F).

**Figure 7:**
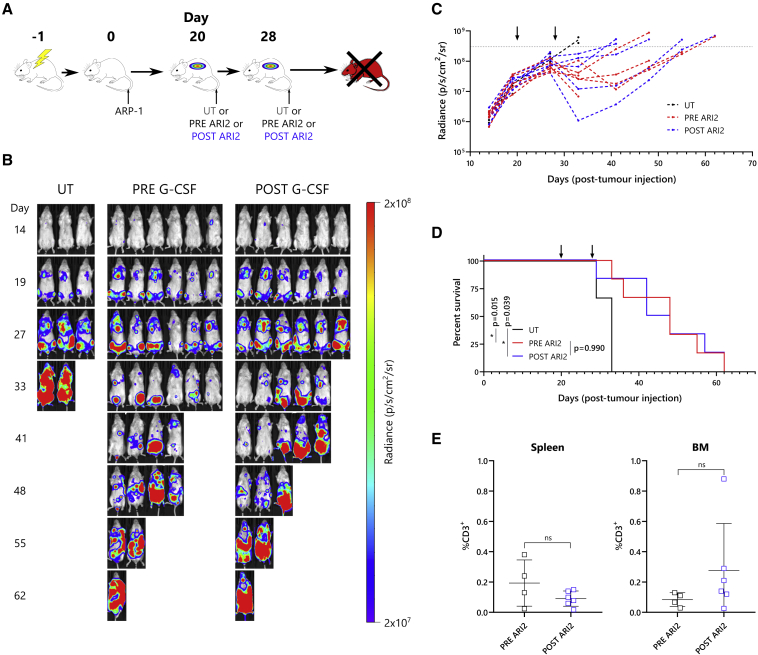
PRE and POST G-CSF ARI2h cells are similarly effective in a mouse MM model (A) Schematic of *in vivo* experimental design. On day 0, irradiated mice were injected with 1.5 × 10^6^ GFP-ffLuc-expressing ARP-1 cells. Following tumor engraftment, mice were transfused twice with UT T cells, PRE G-CSF ARI2h cells, or POST G-CSF ARI2h cells, on day 20 and on day 28. (B) Tumor progression was monitored by weekly readings of animal bioluminescence. Shown are photos taken from the ventral side of the mice. Numbers on the left of the image show the number of days following tumor cell infusion. See also Figure S6C. (C) Quantification of bioluminescence is shown, showing data from each mouse. The dotted horizontal line indicates the predetermined threshold for mouse sacrifice due to heavy tumor burden. (D) Overall survival of mice from each group is shown. (E) Frequency of anti-human CD3^+^ ARI2h cells in the spleen and bone marrow (BM) of mice immediately following sacrifice is shown. Error bars show mean ± SD. Arrows in (C) and (D) indicate days on which T cells were injected. Data points in (E) are coded according to patient donor and are consistent with other figures. ∗p < 0.05; ns, not significant.

To better examine the relative efficacies of PRE and POST ARI2h cells, they were tested in a xenograft mouse model of MM with stress conditions to severely challenge anti-BCMA CAR-T cells. For this experiment, irradiated mice were engrafted with firefly luciferase (ffLuc)-expressing ARP-1 cells until they developed a substantial tumor burden, after which they were dosed with PRE or POST ARI2h cells or UT T cells as a control (Figure 7A). Whereas the animals given PRE or POST ARI2h cells exhibited a decrease or stabilization in disease severity upon T cell administration, those injected with UT cells rapidly developed terminal levels of cancer (Figure 7B, day 33). Analysis of tumor-cell bioluminescence in the following weeks demonstrated that the disease developed similarly in mice treated with PRE or POST ARI2h cells (Figures 7B, 7C, and S6). In turn, there was no difference in either the survival time of animals from the PRE or POST groups (p = 0.990) (Figure 7D) or the persistence of PRE or POST ARI2h cells (Figure 7E).

Overall, these data show that the activity of ARI2h cells is unaffected by previous exposure to G-CSF.

### G-CSF treatment does not negatively impact the *ex vivo* phenotype of expanded ARI2h cells

**Figure 8:**
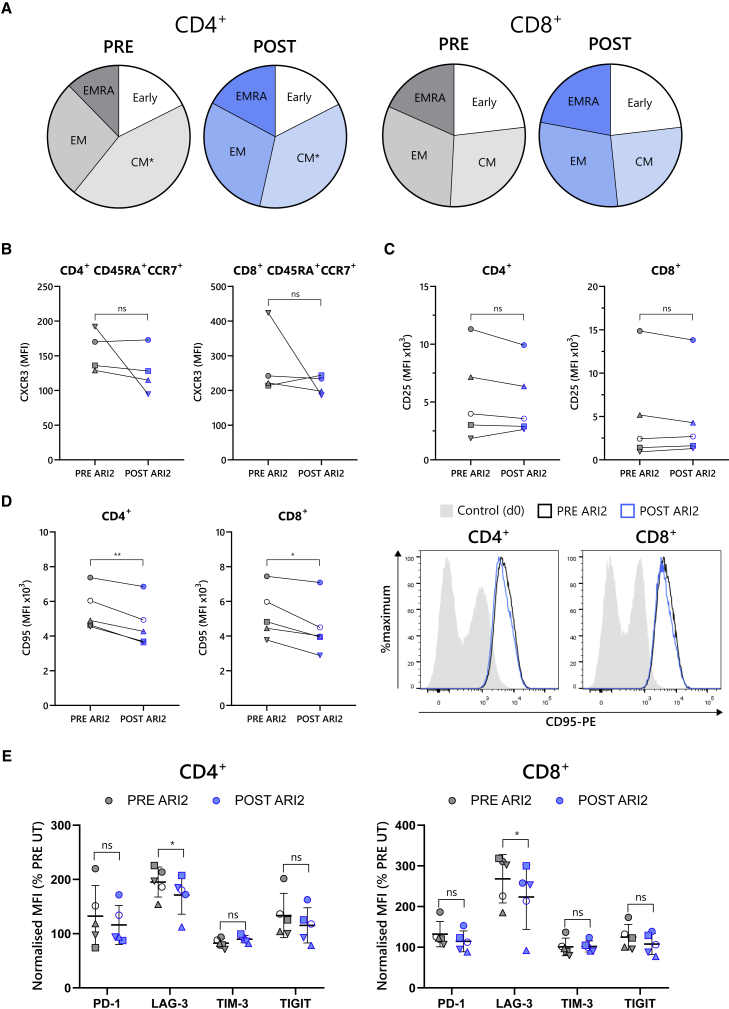
POST G-CSF ARI2h cells have lower expression of LAG-3 and CD95 (A) Summary of memory and effector phenotypes of CD4^+^ (left) and CD8^+^ (right) ARI2h CAR^+^ T cells, categorized as early differentiated (early; CCR7^+^CD45RA^+^), CM (CCR7^+^CD45RA^−^), EM (CCR7^−^CD45RA^−^), and effector memory CD45RA^+^ (CCR7^−^CD45RA^+^) cells. No populations were significantly different in PRE G-CSF ARI2h and POST G-CSF ARI2h cells, with the exception of the CD4^+^ CM subset (p = 0.018). Data represent the mean percentage of each population. n = 4. (B) CXCR3 expression on early differentiated (CCR7^+^CD45RA^+^) CAR^+^ CD4^+^ (left) and CD8^+^ (right) ARI2h cells is shown, based on the MFI of the staining. (C) Measurement of CD25 expression on CAR^+^ CD4^+^ (left) and CD8^+^ (right) ARI2h cells is shown, displayed as MFI. (D) Relative expression of CD95 on CAR^+^ CD4^+^ (left) and CD8^+^ (right) ARI2h cells is shown. Presented is a summary of the CD95 MFI (left) and representative histograms of the staining (right), which includes unstimulated day 0 T cells as a control. (E) Summary of the surface expression of PD-1, LAG-3, TIM-3, and TIGIT on CAR^+^ CD4^+^ (left) and CD8^+^ (right) ARI2h cells is shown, represented as MFI normalized to that displayed on UT PRE G-CSF cells. Gray shapes indicate data from PRE ARI2h cells, and blue shapes represent data from POST ARI2h cells. Error bars show mean ± SD. Data points in (B)–(E) are coded according to patient donor and are consistent with other figures. ∗∗p < 0.01; ∗p < 0.05.

To further explore how G-CSF impacts parameters that could influence the activity of ARI2h cells in patients, the phenotypes of PRE and POST ARI2h cells were analyzed. First, the differentiation status of ARI2h cells from PRE and POST cultures was determined by CD45RA and CCR7 staining. The proportion of each memory and effector cell type was alike, with the only exception being the 20% reduction in the CD4^+^ central memory pool (p = 0.018) (Figure 8A). Likewise, CXCR3 staining of early differentiated CD45RA^+^CCR7^+^ cells, which indicates the presence of T_SCM_-like cells,^37^ was not altered (Figure 8B). Analysis of activation markers demonstrated that CD25 was expressed at similar levels on PRE and POST ARI2 h cells (Figure 8C), but CD95 was significantly reduced on both CD4^+^ and CD8^+^ ARI2h cells within the POST culture (p = 0.003 and p = 0.011, respectively) (Figure 8D). Similar to CD95, expression of the checkpoint molecule LAG-3 was lower on POST ARI2h cells (p = 0.034 and p = 0.035 on CD4^+^ and CD8^+^ cells, respectively), but other exhaustion markers PD-1, TIM-3, and TIGIT were unchanged (Figures 8E and S7).

Together, these results show that the phenotype of POST ARI2h cells is suitable for therapeutic use and, moreover, confers some potential advantages, such as lower LAG-3 expression.

## Discussion

In this study, the functionality of ARI2h cells that had been exposed to G-CSF prior to *ex vivo* expansion was analyzed, with the aim of determining whether these T cells could be used as a starting material for the production of CAR-T cells. After demonstrating that G-CSF had no impact on the activity of ARI2h cells when added *in vitro*, we examined the effect of G-CSF on patient T cells and the ARI2h cells derived from them. We observed that, in this context, G-CSF did not alter T cell characteristics or populations, with the exception of reducing the T_SCM_ pool. However, despite this potential drawback, ARI2h cells displayed no signs of inferiority when produced from T cells that had previously been exposed to G-CSF. This included no augmentation in the expression of checkpoint molecules, alteration in memory and effector populations, or loss of efficacy, either *in vitro* or in a murine xenograft model.

Before analyzing CAR-T cells, we examined the effects of G-CSF on T cells to ensure that they were suitable for CAR-T cell production. Incubation of T cells with recombinant G-CSF excluded most direct influences of G-CSF on T cell phenotype and CAR-T cell function, although it did cause a reduction in ARI2h IL-2 production and TIM-3 expression on CD8^+^ cells. Moreover, we cannot rule out a more general role of G-CSF on T cell function, as this study only evaluated the anti-tumor activity of CAR-T cells in response to MM cells. Indeed, a direct role of G-CSF on T cell effector function has been demonstrated in the context of anti-viral responses.^23^^,^^24^

It was important to study the effect of G-CSF *in vivo*, as well as *in vitro*, because G-CSF-mediated regulation of other immune cell types, such as dendritic cells,^40^ monocytes,^41^ and myeloid-derived suppressor cells (MDSCs),^42^ also causes T cell suppression.^33^ For example, increased MDSC mobilization to the peripheral blood promotes abnormalities in the T cell compartment, such as reduced proliferation and polarization of CD4^+^ cells from a Th1 phenotype towards a Th2 phenotype.^43^^,^^44^ Moreover, the increased presence of histocompatibility leukocyte antigen DR isotype (HLA-DR)^−/low^CD33^+^CD16^−^ MDSCs in peripheral blood causes T reg expansion,^44^ highlighting the complex interplay between G-CSF and a wide range of immune cells. From our results, however, we did not observe any negative effects on T cell phenotype or proliferation that could have occurred as a result of the indirect action of G-CSF on T cells via MDSCs or other cell types. Indeed, T cell proliferative responses and patient T reg numbers were normal after G-CSF infusion. This discrepancy with previously published data could be due to the effects of MM or induction therapy on the immune system, thus dampening any potential effect of G-CSF, or possibly because the POST G-CSF samples were collected immediately after G-CSF administration following only a short exposition to the drug, meaning that any effect of G-CSF had not yet materialized. Supporting this hypothesis, Toh et al.^45^ showed that, during the G-CSF dosage period, Treg frequency was the same as in control samples, but 1–3 weeks later, the percentage of Tregs was significantly higher. Another important difference between our study and that of some other groups is that we used highly purified T cells as a starting material for the *ex vivo* culture of the CAR-T cells and not peripheral blood mononuclear cells (PBMCs). This approach matches the clinical protocol used at our institution^31^^,^^46^ and confers the major advantage of removing T cell inhibitory cell types, such as CD14^+^ monocytes, that severely hinder CAR-T cell expansion.^29^^,^^30^

From all three factors that have been linked with the *in vivo* effectiveness of CAR-T-BCMA cells and patient responses, which are (1) the frequency of CD8^+^ early differentiated cells, (2) the CD4:CD8 ratio of the pre-manufactured T cells, and (3) the *in vitro* expansion rate of the CAR-T cells,^19^ all were unaltered by G-CSF. Early differentiated cells include both naive memory cells and T_SCM_ cells, both of which are believed to have unique roles in the durability of T-cell-based immunotherapies.^47^ Here, we showed that, while the number of CD8^+^ naive cells (CD45RA^+^CCR7^+^CD95^−^ or CD45RA^+^CD27^+^) was unchanged by G-CSF, CD8^+^ T_SCM_ cells (CD45RA^+^CCR7^+^CD95^−^) were diminished. Although the proportion of T_SCM_ in the final CAR-T cell product is correlated with patient responses,^48^ it is currently unclear whether the proportion of T_SCM_ cells in unstimulated T cells influences anti-BCMA CAR-T cell therapy. However, ARI2h cells made from G-CSF-treated T cells had equal *in vivo* function to those not exposed to G-CSF, suggesting that the level of reduction of the T_SCM_ pool caused by G-CSF is insignificant for anti-tumor efficacy. What is more, CXCR3 expression in the CD45RA^+^CCR7^+^ population was the same in PRE and POST ARI2h cells, which indicates similar frequencies of T_SCM_-like cells in the final CAR-T cell product. Another notable property of the infusion CAR-T cell product that was also unaffected by patient G-CSF administration is the CD4:CD8 ratio, which was found to be important for the efficacy of some CAR-T cell products,^49^^,^^50^ but not others.^19^^,^^38^

Another T cell intrinsic quality that is believed to affect CAR-T cell efficacy is dysfunction. In fact, studies of CD19-directed CAR-T cells found that the exhaustion profile of pre-infusion CAR-T cells also correlated with patient responses.^38^^,^^51^ For ARI2h, lower expression of checkpoint inhibitory molecules appears to correlate with activity as well.^52^^,^^53^ Our data revealed that the expression of the checkpoint receptor LAG-3 was lower in POST ARI2h CD8^+^ cells compared with PRE ARI2h CD8^+^ cells, and importantly, the levels of other exhaustion markers (PD-1, TIM-3, and TIGIT) were not increased. LAG-3 expression on T cells appears to be particularly important for MM, because it is associated with event-free survival.^54^ Indeed, in our previous study, we discovered that the IL-15-cultured ARI2h cells that performed better *in vivo* also had lower levels of LAG-3 prior to infusion,^52^ suggesting a link between ARI2h activity and LAG-3 expression.

The next stage to ensure the clinical translation of this study would be to validate the use of frozen and stored apheresis products as a viable option for CAR-T cell generation instead of fresh blood, as was used in this study. Moreover, further investigation is required into the intrinsic qualities that cause T cells from patients early in their MM evolution to be more favorable for CAR-T cell production than those from R/R and heavily treated patients. We plan to address these issues in a novel line of investigation in which the use of frozen and stored mobilized apheresis products collected for ASCT will be compared with peripheral blood drawn from R/R MM patients (i.e., current clinical practice) as a starting material for CAR-T cell production.

Overall, investigations to improve the starting material for anti-BCMA CAR-T cell production, such as this study, are timely due to the substantial number of anti-BCMA CAR-T clinical trials that are ongoing and due to the relevance of our results to the production of all types of CAR-T cells. In fact, an additional advantage to using apheresis products collected for ASCT would be to decrease the burden of apheresis units that are now highly saturated since they must cater to both ASCT and the collection of material for CAR-T cell manufacture. To conclude, we believe that we have provided strong evidence to support the incorporation of apheresis-derived T cells as a starting material for CAR-T cell production in the clinic.

## Materials and methods

### Human samples

MM patients who were in line to receive ASCT were given recombinant G-CSF at a dose of 10–12 μg/kg/12 h for a total of 4 days to facilitate the collection of CD34^+^ progenitor cells by apheresis. Immediately before the first G-CSF dose and shortly after the final G-CSF administration just before the apheresis collection, blood was drawn from patients into EDTA-coated tubes. Patients did not receive plerixafor before blood collection. Buffy coats from healthy donors were obtained from the local blood and tissue bank (Banc de Sang i Teixits, Catalonia). T cells were isolated from fresh blood or buffy coats by density-gradient centrifugation using Histopaque-1077 (Sigma-Aldrich, St. Louis, MO) followed by negative selection of T cells using a Pan T Cell Isolation kit (Miltenyi Biotech, Bergisch Gladbach, Germany). Monocytes were purified from buffy coats using the RosetteSep Human Monocyte Enrichment Cocktail (STEMCELL Technologies, Vancouver, Canada).

### Ethical approval

Informed written consent in compliance with the Declaration of Helsinki was obtained from all donors. Research involving human-derived material and mice was approved by the Ethical Committees of Clinical Research (Hospital Clínic, Barcelona) and Animal Research (University of Barcelona, Barcelona), respectively.

### Cell culture and T cell transduction

T cells were stimulated with Dynabeads Human T-Activator CD3/CD28 (Thermo Fisher Scientific, San Diego, CA) and were expanded in T cell media (47.5% Click’s media [Irving Scientific, Santa Ana, CA], 47.5% RPMI-1640, 5% human serum, 2 mM L-glutamine, 100 IU/mL penicillin, and 100 μg/mL streptomycin) supplemented with 100 IU/mL IL-2. Forty-eight hours following stimulation, T cells were transduced with a lentivirus vector encoding ARI2h.^31^ Subsequently, T cells were split and cytokines were refreshed every 1 to 2 days for a further 7 days of culture, unless indicated otherwise. For experiments in which T cells were exposed to G-CSF in culture, 10 ng/mL recombinant G-CSF (Miltenyi Biotech) was added to T cells that were resting in T cell media for 3 days prior to stimulation. ARP-1, U266, ARP-1-GFP-ffLuc, and U266-GFP-ffLuc cell lines were obtained, cultured, and modified to express GFP-ffLuc, where appropriate, as previously described.^31^ All cultured cells were incubated at 37°C with 5% CO_2_. Live cells were routinely counted using Trypan blue exclusion.

### Flow cytometry

Cell surface proteins on (CAR-)T cells were stained with the following antibodies: annexin V-PE, CCR7-PerCP/Cy5.5, CD3-allophycocyanin (APC), CD3-BV421, CD4-APC/H7, CD8a-PE/Cy7, CD25-PE/Cy7, CD69-fluorescein isothiocyanate (FITC), CD95-PE, CD127-PerCP/Cy5.5, CXCR3-AF488 (all from BD Biosciences, San Jose, CA); CD8a-APC, CD8a-FITC, CD27-PE, CD28-FITC, CD45RA-APC, LAG-3-PE, TIGIT-PerCP/Cy5.5, TIM-3-FITC (all from BioLegend, San Diego, CA); and PD-1-APC (eBioscience, Thermo Fisher Scientific). Monocytes were stained with CD14-PE (eBioscience). To determine CAR expression, cells were labeled with a recombinant Fc-tagged BCMA protein (Enzo Life Sciences, Farmingdale, NY) followed by a BV421-conjugated anti-Fc antibody (BioLegend). Samples prepared from mouse organs were treated with Fc block (BD Biosciences) prior to staining. Cells were washed and resuspended in fluorescence-activated cell sorting (FACS) buffer (1% FCS in PBS) or 1% paraformaldehyde prior to acquisition. In experiments to assess viability and/or apoptosis, cells were resuspended in FACS buffer containing 1 μg/mL 7-aminoactinomycin D (7-AAD). In all other experiments, live cells were gated based on forward and side scatter.

All flow cytometry data were acquired on a FACSCanto II (BD Biosciences) and analyzed using FlowJo software version X.0.7 (Treestar, Ashland, OR).

### Staining of Tregs

Following cell surface staining, T cells were fixed and permeabilized using the Foxp3/Transcription Factor Staining Buffer Set (eBioscience) according to the manufacturer’s instructions. During the permeabilization step, Tregs were stained with a PE-conjugated FoxP3 antibody (BD Biosciences). Tregs were identified as CD4^+^CD25^high^FoxP3^+^ cells with conventional CD4^+^ T cells used as a negative control (full gating strategy shown in Figure S8).

### Analysis of granzyme B expression

Equal numbers of ARI2h and ARP-1 cells were co-cultured for 6 h at 37°C, with GolgiPlug (BD Biosciences) added at a dilution of 1:1,000 after the 1^st^ hour. The cells were then stained for surface markers, fixed, and permeabilized according to the instructions of the FIX&PERM Cell Fixation and Permeabilization Kit (Nordic MU Bio, Susteren, the Netherlands) with the inclusion of an AF647-conjugated anti-granzyme B antibody (BD Biosciences) during the permeabilization stage.

### *In vitro* cytotoxicity assay

To assess ARI2h anti-tumor cell activity, an *in vitro* luminescence-based assay was employed, as previously described.^50^ In brief, GFP-ffLuc-expressing ARP-1 (ARP-1-GFP-ffLuc) or U266 (U266-GFP-ffLuc) cells were co-cultured with ARI2h cells at the indicated effector:target ratios. D-luciferin was added to the cells 10 min prior to analysis, and the luminescence signal was read on a plate reader. ARI2h activity was interpreted based on the luminescence signal emitted by the surviving tumor cells, which was calculated as follows:

$$100\times \frac{\left(signal\;from\;sample\;well-background\;signal\right)}{\left(signal\;from\;well\;containing\;tumour\;cells\;alone-background\;signal\right)}$$

### ELISAs

ARI2h cells and ARP-1 or U266 cells were co-cultured for 6 h at a 1:1 ratio, after which the supernatants were collected and stored at −80°C until analysis. The concentrations of IFNγ and IL-2 in the supernatants were analyzed by ELISA using commercially available kits (BioLegend).

### *In vivo* murine experiments

Mouse experiments were performed similarly to those described previously.^52^ In detail, on day 0, 1.5 × 10^6^ ARP-1-GFP-ffLuc cells were injected into the tail vein of irradiated 9-week-old female non-obese diabetic (NOD)-severe combined immunodeficiency (SCID)-IL-2gc^−/−^ mice, and on day 20 post-tumor injection, the mice received 8.6 × 10^6^ UT T cells or 6 × 10^6^ CAR^+^ PRE or POST ARI2h cells (8.6 × 10^6^ total cells) through intravenous inoculation. Eight days later, on day 28, the mice received a second dose of CAR-T cells: 2.9 × 10^6^ UT T cells or 2 × 10^6^ CAR^+^ PRE or POST ARI2h cells (2.9 × 10^6^ total cells). Every week, beginning on day 7, tumor growth in each mouse was monitored by injection with D-luciferin and measurement of the bioluminescence signal on both the ventral and dorsal sides. The signals were averaged, and when the mean bioluminescence exceeded 3 × 10^8^ p/s/cm^2^/sr, mice were sacrificed. The spleen and the bone marrow from the hind legs of sacrificed animals were used to harvest cells and prepare FACS samples. Mice were assigned to each group randomly, and the experiment was performed in a blinded manner so that both the investigator analyzing tumor burden and the technician injecting the T cells into the mice were unaware which mouse received which T cell treatment.

### Proliferation measurements

ARI2h cells were starved of IL-2 for 24 h and then stained with carboxyfluorescein succinimidyl ester (CFSE) following a published protocol.^55^ CFSE-stained ARI2h cells were then cultured with ARP-1 cells for 3 days at a 0.5:1 ratio of ARI2h:ARP-1 cells and analyzed by flow cytometry. Control cultures included ARI2h cells cultured alone, with or without IL-2, and co-cultures that included ARI2h cells that were not stained with CFSE.

### Repeated stimulation of ARI2 h cells with tumor cells

We co-cultured 0.125 × 10^6^ ARI2h cells with 1 × 10^6^ ARP-1-GFP-ffLuc cells in a total of 2 mL for 72 h. Thereafter, stimulated ARI2h cells were combined with 1 × 10^6^ fresh ARP-1-GFP-ffLuc cells at the same E:T ratio (0.125:1). ARI2h cells were restimulated with ARP-1-GFP-ffLuc cells three times (a total of four rounds of stimulation). Following each round of stimulation, ARI2h cells were counted using an Attune NxT flow cytometer (Thermo Fisher Scientific). Every 24 h, aliquots of the co-cultures were removed to quantify tumor cell survival, measured as described above.

### Immunoblotting

Monocytes were resuspended in RPMI-1640 media supplemented with 10% fetal calf serum (FCS) at 2.5 × 10^6^/mL and incubated with 1–100 ng/mL recombinant G-CSF or vehicle control for 10 min at 37°C. Cell stimulation was halted by the addition of ice-cold PBS and rapid centrifugation. After an additional wash in PBS, pellets were frozen and stored at −80°C. Cells were lysed in buffer (20 mM Tris [pH 7.4], 150 mM NaCl, 1% Triton X-100, 0.01% SDS, 1 mM EDTA, 1 mM phenylmethylsulfonyl fluoride, and 1 mM Tris(2-carboxyethyl)phosphine [TCEP]) supplemented with 1% Protease Inhibitor Cocktail, 1% Phosphatase Inhibitor Cocktail 2, and 1% Phosphatase Inhibitor Cocktail 3 (all Sigma-Aldrich) for 10 min at 4°C before centrifugation. Clarified lysates were then supplemented with Laemmli Sample Buffer (Bio-Rad) containing 25 mM TCEP and boiled for 5 min. Samples were resolved by SDS-polyacrylamide gel electrophoresis using 10% Mini-PROTEAN TGX Precast Protein Gels (Bio-Rad, Hercules, CA), and proteins were transferred to polyvinylidene fluoride (PVDF) membrane. Blots were probed with primary antibodies targeting pSTAT3^Y705^ (Cell Signaling Technology, Danvers, MA) or β-actin (Santa Cruz Biotechnologies, Dallas, TX) before incubation with horseradish-peroxidase-conjugated secondary antibodies (goat anti-rabbit [Cell Signaling Technology] or goat anti-mouse [LI-COR Biosciences, Lincoln, NE]). Membranes were exposed to SuperSignal West Pico Chemiluminescent Substrate (Thermo Fisher Scientific), and the resulting chemiluminescence signal was visualized on an ImageQuant LAS 4000 Mini system (GE Healthcare, Chicago, IL). To detect total STAT3 levels, membranes initially used to blot for pSTAT3^Y705^ were stripped with a mild stripping buffer (0.2 M glycine [pH 2.5], 0.1% SDS, and 0.1% Tween-20) and reprobed with an anti-STAT3 antibody (Cell Signaling Technology).

### Statistical analysis

GraphPad Prism v.8.0.1 (GraphPad Software, San Diego, CA) was used for data analysis. For *in vitro* experiments, comparisons of two groups were performed using two-tailed paired Student’s t tests, and multiple comparisons were conducted using a repeated-measures one-way ANOVA corrected with a Tukey post-hoc test. Comparison of mouse survival between different groups was analyzed using the log rank (Mantel-Cox) test, and differences in the presence of ARI2h cells in murine organs was assessed using a two-tailed unpaired Student’s t test.

## Data availability

Data are available upon reasonable request.

## Supplemental information

Document S1. Figures S1–S8 and Tables S1 and S2

Document S2. Article plus supplemental information
