# Supplementary material for: T cells isolated from G-CSF-treated multiple myeloma patients are suitable for the generation of BCMA-directed CAR-T cells
Source: Mol Ther Methods Clin Dev. 2022 Jun 22;26:207–23. doi: 10.1016/j.omtm.2022.06.010 (PMC9271987; doi:10.1016/j.omtm.2022.06.010)
Supplement: Document S1. Figures S1–S8 and Tables S1 and S2 [file mmc1.pdf]

OMTM, Volume *26*

Supplemental information

T cells isolated from G-CSF-treated multiple myeloma patients are suitable for the generation of BCMA-directed CAR-T cells

Anthony M. Battram, Aina Oliver-Caldés, Maria Suárez-Lledó, Miquel Lozano, Miquel Bosch i Crespo, Núria Martínez-Cibrián, Joan Cid, David F. Moreno, Luis Gerardo Rodríguez-Lobato, Alvaro Urbano-Ispizua, and Carlos Fernández de Larrea

Table S1. Characteristics of multiple myeloma patients (n = 8).

Parameter	Value, n
Median age (range), years	62 (44-66)
Sex	
Male	1
Female	7
ISS[a]	
I	1
II	5
III	2
R-ISS[b]	
I	1
II	2
III	5
Isotype	
IgG	5
IgA	1
Light chain only	2
Cytogenetics[c]	
Del17p	3
t(11;14)	1
IGH rearrangement	2
Hyperdiploidy	1
No adverse cytogenetics	3
Median bone marrow plasma cell infiltration (range), %	53 (16-85)
Extramedullary disease	
Yes	0
No	8
Median creatinine at diagnosis (range), mg/dL	0.74 (0.56-1.01)
Lines of previous therapy	
1	8
Stem cell mobilisation (days)	
4	8

[a]ISS, International Staging System

[b]R-ISS, Revised International Staging System

[c]n>8 as some patients had multiple adverse cytogenetic characteristics

Table S2. Induction therapy (n = 8).

Parameter	Value, n
Induction therapy regimen[a]	
VRD	2
KRD	6
Response to therapy[b]	
VGPR	4
CR	4
Cycles of therapy	
4	5
5	3

[a]VRD, bortezomib/lenalidomide/dexamethasone; KRD, carfilzomib/lenalidomide/dexamethasone

[b]VGPR, very good partial response; CR, complete response

Figure S1

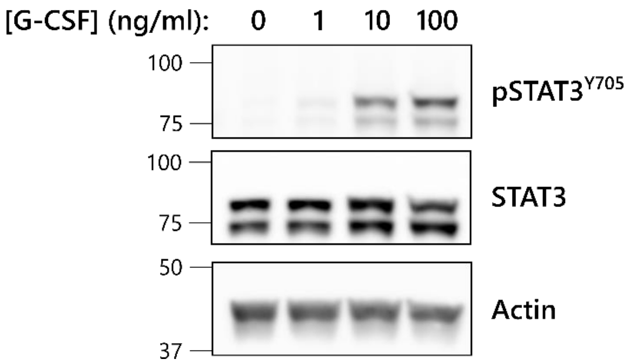

Figure S1. Recombinant G-CSF induces STAT3 phosphorylation in monocytes Monocytes treated with 0-100 ng/ml recombinant G-CSF for 10 minutes were subject to Western blotting to assess STAT3 phosphorylation. Membranes were reprobed for total STAT3 and β-actin was used as a loading control. Numbers on the left of the blots show molecular weights in kDa. Results are representative of 2 independent experiments.

Figure S2

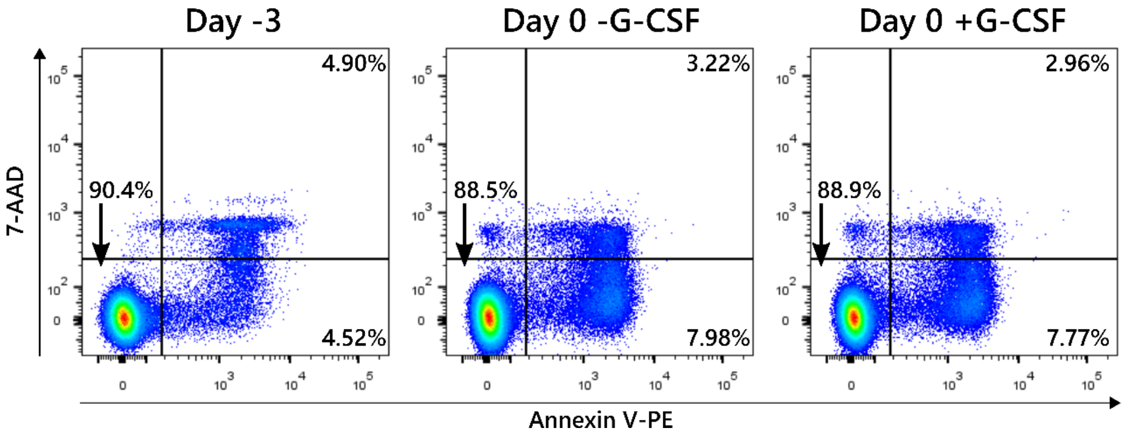

Figure S2. Staining of T cells to assess apoptosis Cells were stained with annexin V (AnV) and 7-AAD on day -3 before exposure to G-CSF and afterwards on day 0 to determine the frequency of live cells (AnV-7-AAD-), early apoptotic cells (AnV+7-AAD-) and late apoptotic/dead cells (AnV+7-AAD+). Numbers indicate frequency of each population compared to total cells. See also Figure 1A-B.

Figure S3

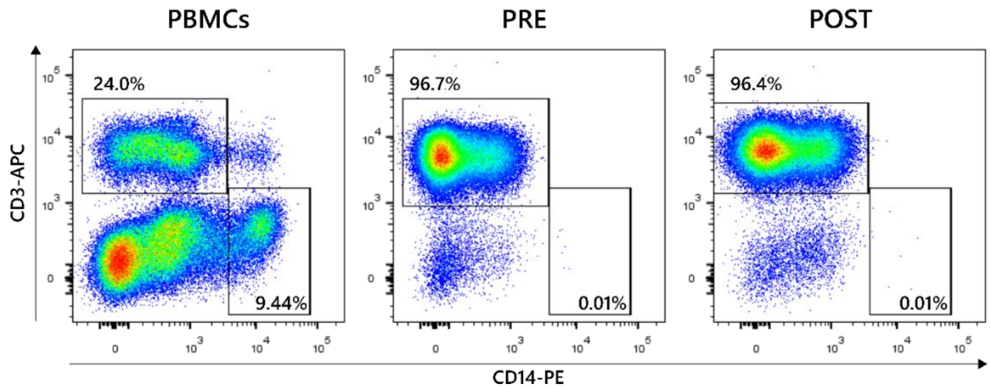

Figure S3. T-cell isolation from PBMCs successfully removes monocytes T cells freshly isolated from patients before (PRE) and after (POST) G-CSF administration were analysed for the presence of T cells and monocytes by staining for CD3 and CD14, respectively. PBMCs from POST G-CSF samples before T-cell isolation were also stained as a control. Numbers indicate frequency of each population compared to total cells.

Figure S4

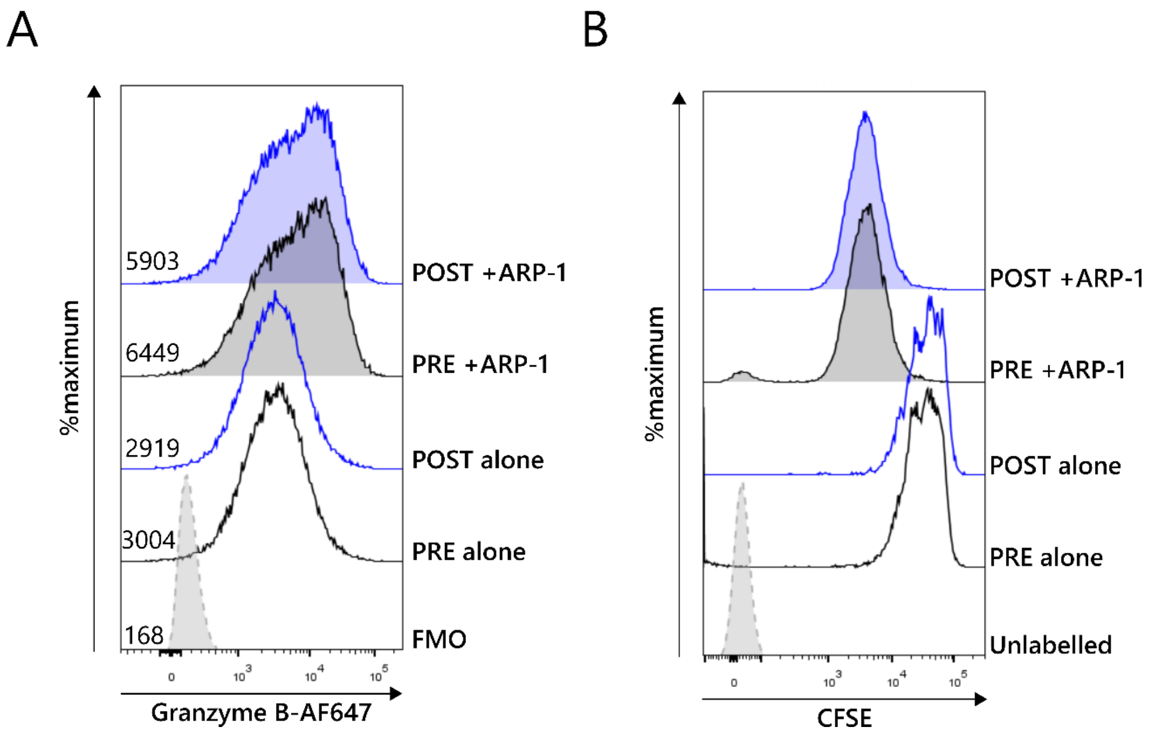

Figure S4. CD8⁺ ARI2h cell granzyme B production and proliferation of CD4⁺ ARI2h cells in response to myeloma cells are unaffected by G-CSF exposure (A) UT and ARI2h cells were co-incubated with ARP-1 cells or media alone (alone) for 6 hours. Expression of granzyme B in $CD8^+$ CAR^+ cells was assessed by flow cytometry and displayed are histograms representative of 3 experiments. FMO indicates fluorescence minus one (fully stained except for granzyme B) control and numbers on histograms show MFI. See also Figure 6D. (B) ARI2h cells were labelled with CFSE and cultured with (+ARP-1) or without (alone) ARP-1 cells. After 72 hours, ARI2h cell proliferation was analysed by measuring CFSE dilution. ARI2h cells that were not treated with CFSE (Unlabelled) acted as a control. The displayed histograms are $CD4^+$ CAR^+ cells and are representative of 3 experiments. See also Figure 6E.

Figure S5

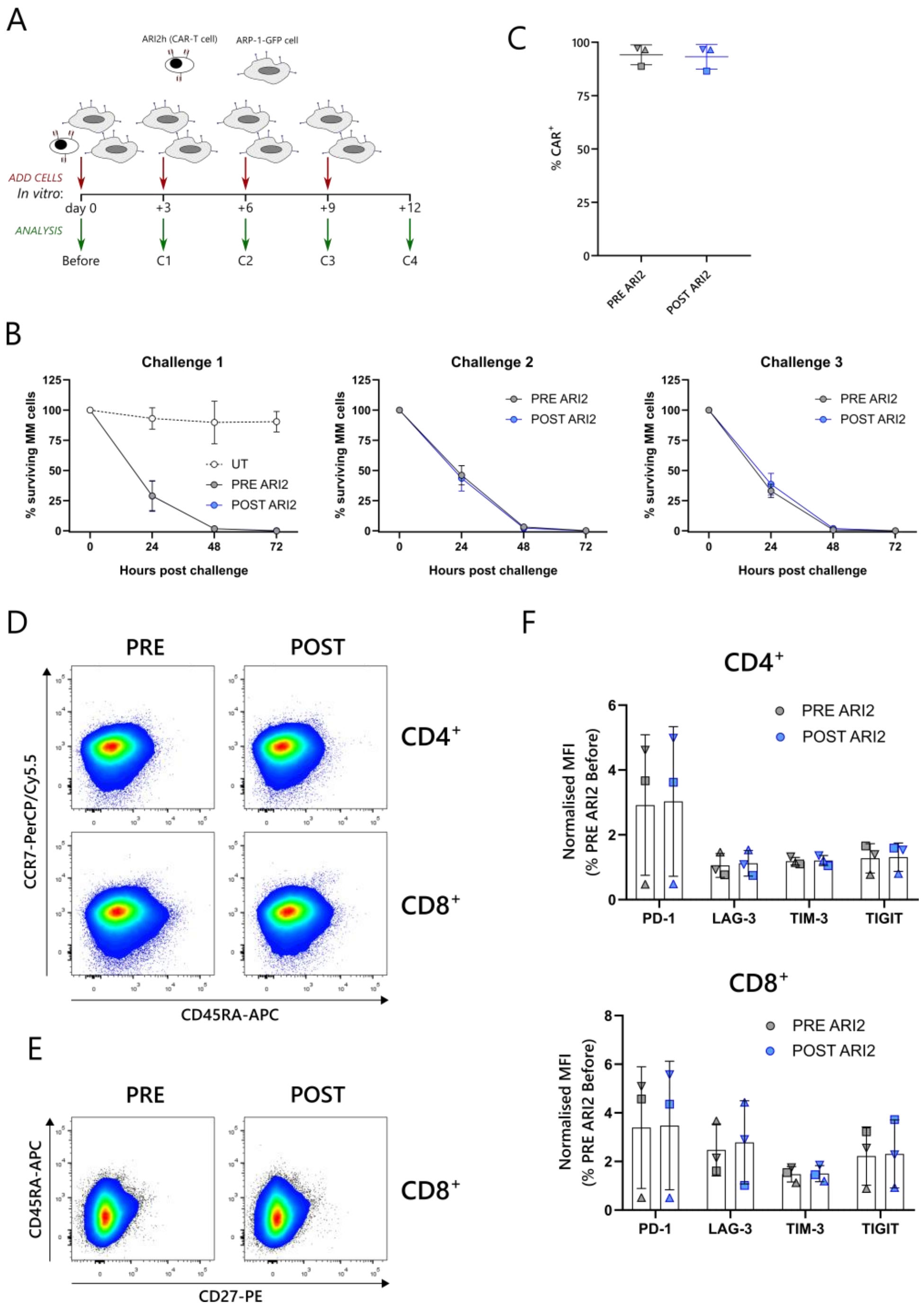

Figure S5. PRE G-CSF ARI2h and POST G-CSF ARI2h cells respond similarly to repeated stimulation with tumour cells 0.125x10^{6} ARI2h cells were mixed with 1x10^{6} GFP-ffLuc-expressing ARP-1 (ARP-1-GFP) cells. 72 hours later (day +3), ARI2h cells were counted and combined with fresh ARP-1-GFP cells at the same ARI2h cell:ARP-1-GFP cell ratio (0.125:1). This tumour cell stimulation was performed a total of four times. See also Figure 6F. (A) Schematic of *in vitro* repeated stimulation experimental design. (B) Survival of ARP-1-GFP cells during the first (Challenge 1), second (Challenge 2) and third (Challenge 3) stimulations, determined by measuring target cell bioluminescence

(n = 3). Data are represented as mean ± SEM. See also Figure 6G. (C-F) Percentage of T cells that were positive for the ARI2h CAR molecule (C), CCR7/CD45RA staining of $CD4^{+}$ and $CD8^{+}$ CAR^{+} cells (D), CD27/CD45RA staining of $CD8^{+}$ CAR^{+} cells (E) and summary of exhaustion marker expression (F) after all four stimulations. FACS plots in panels D and E are representative of 3 experiments and data points in panel F are coded according to patient donor and are consistent with other figures. Error bars show mean ± SD.

Figure S6

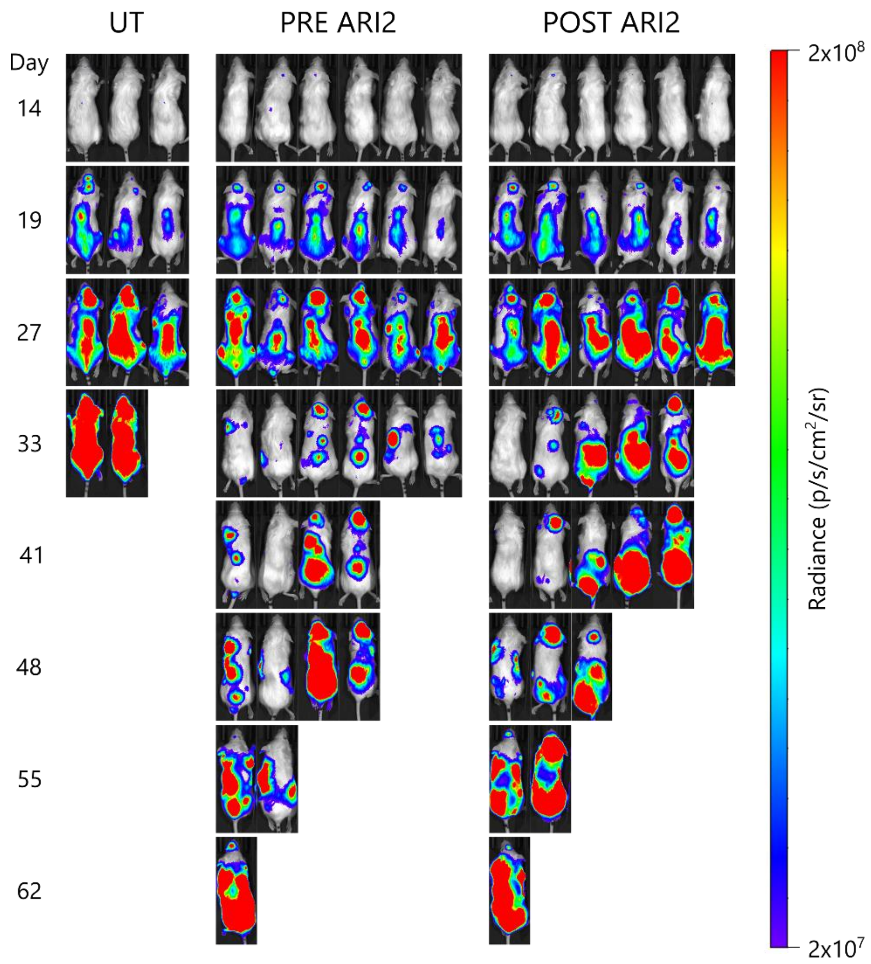

Figure S6. PRE and POST G-CSF ARI2h cells are similarly effective in a mouse MM model *In vivo* models were performed as shown in Figure 7A. Tumour progression was monitored by weekly readings of animal bioluminescence. Shown are photos taken from the dorsal side of the mice. Numbers on the left of the image show the number of days following tumour cell infusion. See also Figure 7B.

Figure S7

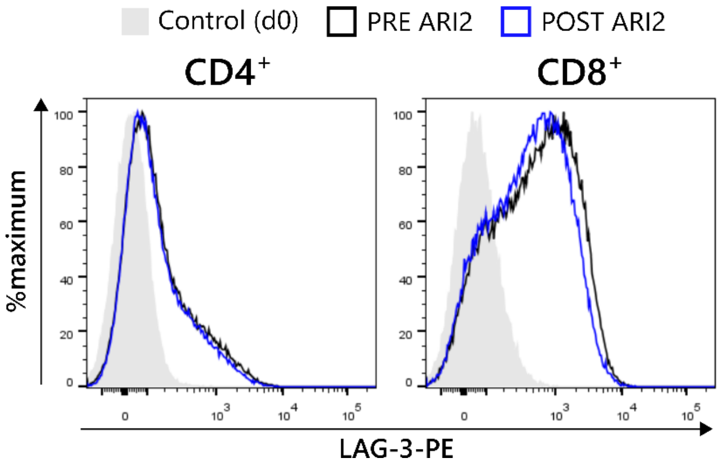

Figure S7. LAG-3 expression is reduced on POST G-CSF ARI2h cells Relative expression of LAG-3 on CAR^+ $CD4^+$ (left) and $CD8^+$ (right) ARI2h cells and unstimulated day 0 T cells as a control. Histograms are representative of 5 experiments. See also Figure 8E.

Figure S8

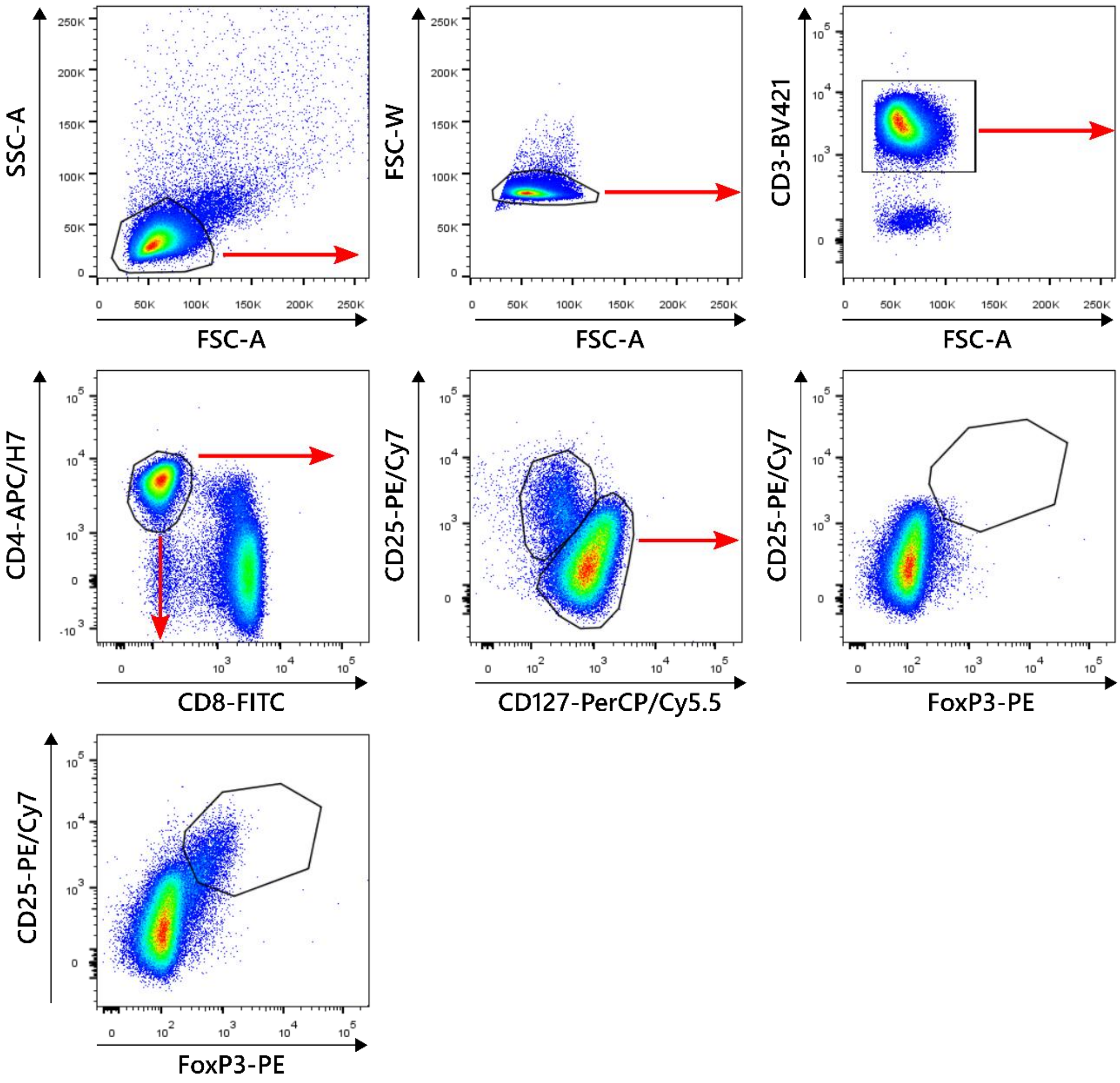

Figure S8. Gating strategy to quantify CD4^{+}CD25^{high}FoxP3^{+} Tregs CD4^{+} conventional T cells (CD25^{low}CD127^{high}) were used to generate a gate to identify CD25^{high}FoxP3^{+} Tregs from the total population of CD4^{+} T cells.
